
JOURNAL INFORMATION
==============================
NLM Title Abbreviation: Inter Econ
Journal ID: Inter Econ
NLM Journal ID: 101086399

Inter Economics

ISSN: 0020-5346

ARTICLE INFORMATION
==============================
PMCID: PMC8853111
PMID: 35194234
DOI: 10.1007/s10272-022-1017-x
Article ID: 1017
Article version: 1
Subjects: Editorial

A Three Percent Structural Deficit Rule

Breuer Christian c.breuer@zbw.eu

grid.461649.8 0000 0001 1019 0408 Intereconomics, ZBW, Neuer Jungfernstieg 21, 20354 Hamburg, Germany
Electronic publication date: 2022 Feb 12
Print publication date: 2022
Volume: 57
Issue: 1
First page: 2
Last page: 3
Copyright: © The Author(s) 2022
License: Open Access: This article is distributed under the terms of the Creative Commons Attribution 4.0 International License (https://creativecommons.org/licenses/by/4.0/).
Open Access funding provided by ZBW — Leibniz Information Centre for Economics.
License URL: https://creativecommons.org/licenses/by/4.0/

Keywords: E61, E62, E63
issue-copyright-statement: © The Author(s) 2022

==============================

Thirty years ago, on 7 February 1992, the Maastricht Treaty was signed. It laid the foundations for the Economic and Monetary Union (EMU). While Germany has been sceptical about a monetary union without a political union, Chancellor Kohl accepted the Delors plan that lead to the EMU in a three-stage process. To compensate for the lack of a political union and to avoid fiscal pressure on the new independent European Central Bank (fiscal dominance), Germany pushed forward strict entry conditions and rules. Particularly the fiscal rules – the 3% deficit and the 60% debt reference values – have been controversial from the beginning (Priewe, 2020). They have become even more controversial since the European debt crisis and the introduction of the Fiscal Compact that intended to achieve close to budget balance medium-term budgetary objectives.

Since the start of the COVID-19 crisis in 2020, the EU's fiscal rules have been suspended, but they are expected to be reinstated after 2022, which is why researchers and policymakers are discussing whether and how the rules should be renegotiated and modernised. According to policymakers, the reform of the Stability and Growth Pact should maintain debt sustainability and ensure growth- and climate-friendly investment. Moreover, the fiscal rules should become simpler and more transparent (German Federal Government, 2021; Draghi and Macron, 2021; Cuerpo, 2022).

The recent discussion takes place against the background of quite remarkable and exceptional macroeconomic conditions in the major OECD economies. In 2021, the newly elected US administration enacted large-scale spending and investment packages that increased deficits, GDP, employment as well as inflation, and will force the Fed to quickly increase the federal funds interest rate this year. These actions will shift the fiscal-monetary policy mix away from expansionary monetary towards fiscal policy. The US has escaped a "Japanese disease". Japan has tried to achieve moderate inflation with expansionary fiscal policy and increasing levels of public debt for decades; however, the country is stuck at the effective zero lower bound, and recent forecasts see inflation at 1.1%, well below the inflation target rate of 2%.

The EMU, with its current fiscal framework, would be forced to push deficits back to close to balanced budgets if no reform takes place next year. Contrary to Japan and the US, the EMU would choose the opposite side of the fiscal-monetary policy mix, i.e. contractionary fiscal policy that the ECB will likely compensate with super expansionary policy, as it has done since the European debt crisis, after the introduction of the Fiscal Compact.

The question of whether the recent low interest rates are a temporary phenomenon or reflect a secular trend is one of the most important macroeconomic controversies today. Von Weizsäcker and Krämer (2021) argue that the natural rate of interest (the risk-free real rate of interest that is compatible with full employment in a closed economy without public debt) can be (and turns out to be) below zero in the OECD countries and China. The theory has important implications for the fiscal framework. To stabilise real interest rates at reasonable levels, von Weizsäcker and Krämer argue that it is necessary to abolish the current fiscal rules, particularly in Germany, and stimulate demand with expansionary fiscal policies. They suggest that countries eliminate their current account surpluses (deficits) in periods of low (high) real interest rates by increasing (decreasing) government net borrowing. This could be an interesting takeaway in the recent debate given that the EU framework already has rules to avoid excessive current account imbalances. It does not, however, put a great deal of emphasis on the rules regarding current accounts. To fill this gap, Mathieu and Sterdyniak (2022) discuss the possibilities of fiscal policy to stabilise current accounts in EMU countries in line with the inflation target.

In line with von Weizsäcker and Krämer (2021), a recent contribution by Blanchard (2022) investigates the determinants of the so-called neutral safe interest rate and argues that the neutral rate decreased since the early 1990s and now turns out to be negative. Blanchard discusses the implications of low safe rates on the sustainability conditions of public debt. If the interest rate r is below the growth rate of the economy g, the government can run a primary budget deficit (i.e. the budget deficit excluding interest payments). Blanchard (2021) argues that no simple fiscal rule would work well under r<g, but if one is to take rules into account, the debt sustainability condition should be used, which says that under a given relation of interest rates and GDP growth (r-g), to balance the debt-to-GDP ratio b at a constant rate, the primary balance should equal (r-g)*b.

Ironically, for the current situation in Europe, this condition would almost exactly deliver the 3% Maastricht deficit rule: under the reasonable assumption of 3% nominal growth (1% real and 2% inflation), approximately 100% of government debt per GDP and any interest rate. Under these conditions, the primary surplus would equal r-3%, which would translate into a general 3% deficit rule, if r is the average rate of interest on government bonds.

The 3% deficit rule has an advantage over even the debt sustainability condition, as suggested by Blanchard (2021). It would give more fiscal space (in terms of primary deficits) to countries with low levels of debt and less space to countries with debt levels larger than 100%. In line with this idea, Regling (2022, 9) indicates that "a simplified and credible EU fiscal framework could be built around a 3% deficit limit".

The good news: first, the EU could keep the most prominent indicator, the 3% deficit limit. That could facilitate the agreement between the two camps, supporters and opponents of fiscal rules.

Second, the EU could keep the instruments to compute the structural budget balance: In order to ensure sustainability and to avoid procyclicality, the 3% reference value could be interpreted as a structural (cyclically adjusted) reference value. The cyclical adjustment strategy was one of the improvements of the second generation budget rules, although if it is far from perfect (Breuer, 2021).

Third, to address the issues related to the estimation of cyclical deficits, as indicated by the new German coalition, the reform could try to improve the methods of computing output gaps (German Federal Government, 2021). That could, for example, take the form of indicators that estimate full-employment output gaps or indicators that take into account deviations from target inflation, real exchange rates or current account imbalances. Giving countries with current account surpluses more fiscal space would contribute to reducing imbalances.

Fourth, the reform could be accompanied by a proposal of how to support investment. This could either be a rule that a certain part of the 3% deficit would be reserved for (net) investments or an additional fund within the national or EU budget reserved for (certain types of) investment.

Christian Breuer, ZBW — Leibniz Information Centre for Economics, Hamburg, Germany.


References

Blanchard, O. (2021, 21 December), Why low interest rates force us to revisit the scope and role of fiscal policy: 45 takeaways, PIIE Realtime Economic Issues Watch.
Blanchard, O. (2022), Fiscal Policy under Low Interest Rates, a draft for open review, MIT Press.
Breuer C Structural Indicators and the Fiscal Uncertainty Principle Intereconomics 2021 56 4 182 183 10.1007/s10272-021-0977-6 34376862 PMC8339685
Cuerpo C Economic Recovery in the Age of COVID-19 Intereconomics 2022 57 1 5 7 10.1007/s10272-022-1019-8 PMC8852929 35194235
Draghi, M. and E. Macron (2021, 23 December), The EU’s fiscal rules must be reformed, Financial Times.
German Federal Government (2021), Mehr Fortschritt wagen: Bündnis für Freiheit, Gerechtigkeit und Nachhaltigkeit, Koalitionsvertrag zwischen SPD, BÜNDNIS 90/DIE GRÜNEN und FDP.
Mathieu C Sterdyniak H Towards New Fiscal Rules in the Euro Area? Intereconomics 2022 57 1 16 20
Priewe J Europäische Wirtschafts- und Währungsunion: Grenzwerte für Defizite und Schulden in der Kritik Wirtschaftsdienst 2020 100 7 538 544 10.1007/s10273-020-2701-8
Regling K EU Fiscal Rules: A Look Back and the Way Forward Intereconomics 2022 57 1 8 10 10.1007/s10272-022-1020-2 PMC8853297 35194236
von Weizsäcker, C. C. and H. Krämer (2021), Saving and Investment in the Twenty-First Century — The Great Divergence, Springer.
